# A Solution Quality Assessment Method for Swarm Intelligence Optimization Algorithms

**DOI:** 10.1155/2014/183809

**Published:** 2014-06-11

**Authors:** Zhaojun Zhang, Gai-Ge Wang, Kuansheng Zou, Jianhua Zhang

**Affiliations:** ^1^School of Electrical Engineering and Automation, Jiangsu Normal University, Xuzhou, Jiangsu 221116, China; ^2^School of Computer Science and Technology, Jiangsu Normal University, Xuzhou, Jiangsu 221116, China

## Abstract

Nowadays, swarm intelligence optimization has become an important optimization tool and wildly used in many fields of application. In contrast to many successful applications, the theoretical foundation is rather weak. Therefore, there are still many problems to be solved. One problem is how to quantify the performance of algorithm in finite time, that is, how to evaluate the solution quality got by algorithm for practical problems. It greatly limits the application in practical problems. A solution quality assessment method for intelligent optimization is proposed in this paper. It is an experimental analysis method based on the analysis of search space and characteristic of algorithm itself. Instead of “value performance,” the “ordinal performance” is used as evaluation criteria in this method. The feasible solutions were clustered according to distance to divide solution samples into several parts. Then, solution space and “good enough” set can be decomposed based on the clustering results. Last, using relative knowledge of statistics, the evaluation result can be got. To validate the proposed method, some intelligent algorithms such as ant colony optimization (ACO), particle swarm optimization (PSO), and artificial fish swarm algorithm (AFS) were taken to solve traveling salesman problem. Computational results indicate the feasibility of proposed method.

## 1. Introduction

Swarm intelligence (SI) optimization [1] is a class of certain population-based metaheuristics which are inspired by the behavior of swarm of agents (i.e., living beings) interacting locally with each other and with their environment. SI is relatively new subfield of artificial intelligence. The behavior of every agent in SI is simple and does not have intelligence. But a number of simple agents through local rules are able to have the emergence of collective intelligence and come to intelligent solutions for complex problems. In recent years, SI has received widespread attention in research. Typical SI schemes include ant colony optimization (ACO) [2], particle swarm optimization (PSO) [3], artificial bee colony (ABC) [4], and artificial fish swarm algorithm (AFS) [5].

ACO is a class of optimization algorithms modeled on the foraging behavior of an ant colony. In ACO, a colony of artificial ants with the artificial pheromone trails and heuristic information are stochastic constructive heuristics that build better and better solutions by using and updating pheromone trail. New solutions are generated using a parameterized probabilistic model, the parameters of which are updated using previously generated solutions so as to direct the search towards promising areas of the solution space. The first ACO algorithm is ant system (AS) [6]. In the next years, many kinds of ACO algorithms have been developed to improve the performance of AS, such as ant colony system (ACS) [7], max-min ant system (MMAS) [8], and two-stage updating pheromone for invariant ant colony optimization algorithm (TSIACO) [9]. PSO is a metaheuristic search method that simulates the movements of a flock of birds which aim to find food. PSO optimizes a problem by having a population of candidate solutions, called particles, and moving these particles around in the search space according to simple mathematical formulae over the particle's position and velocity. Each particle's movement is influenced by its local best known position and also guided toward the best known positions in the search space, which are updated as better positions founded by other particles. The first PSO algorithm was introduced by Kennedy and Eberhart. ABC is an optimization algorithm based on the intelligent foraging behavior of honey bee swarm, proposed by Karaboga in 2005. In the ABC model, the colony consists of three groups of bees: employed bees, onlookers, and scouts. It is assumed that there is only one artificial employed bee for each food source. Employed bees go to their food source and come back to hive and dance on this area. The employed bee whose food source has been abandoned becomes a scout and starts to search for finding a new food source. Onlookers watch the dances of employed bees and choose food sources depending on dances. The scout bee moves in the solution space to discover new food sources. SI has been applied to many applications problems, such as knapsack problems, scheduling problems, assignment problems, multiobjective optimization problem, and cluster analysis.

Although great progress has been achieved in application, there is also a basic question, which is how to quantify the goodness of the solution obtained in finite time, needed to be answered. We call it solution quality evaluation problem. At present, the existing researches focus on the solution “value performance,” namely, the difference between the solution obtained by algorithm and the optimal solution of the problem. The general use of the method is ratio analysis, namely, ratio between solution obtained by algorithm and optimal solution. If the ratio is closer to 1, it means that higher quality is obtained by algorithm and the algorithm is more effective. Competitive analysis [10] of online algorithm also can be employed. The drawback of two methods is that they need optimal solution of problem. There are some approximation methods used to estimate optimal for example, extreme value theory [11] and Lagrange's relaxation method [12], to get the solution value or bound to replace the optimal solution in practical problems. This analysis method generally requires strong theoretical basis of mathematic and strong math skills, and even it is difficult or impossible to give this kind of boundary for most of the problems. In addition to bias in the theoretical study of evaluation methods, some scholars pay more attention to the experimental analysis method. Hoos and Stützle [13] proposed to analyze the performance and behavior of stochastic local search algorithm by experimental analysis method. The performance of several existing particle swarm optimization algorithms was compared by using this method, and an improved particle swarm optimization algorithm was introduced according to the law in [14].

With development of the ordinal optimization (OO) theory [15], the research changes the angle to solution “ordinal performance” to evaluate solution quality of optimization method. Here the solution “ordinal performance” refers to the judgment about whether the solution is belonging to the good enough solution set. Shen et al. [16] used solution comparison between heuristic methods and uniform sampling to evaluate the solution. The evaluation criterion is alignment probability used in OO. As the extension of this work, author used the knowledge of hypothesis testing to develop it into a theory in [17]. In this paper, we proposed an experimental analysis method based on the analysis of search space and characteristic of algorithm itself to evaluate the solution quality for SI.

The rest of this paper is organized as follows: Section 2 reviews the basic idea of OO and indicates the difficulty of quantifying solution quality by analyzing the existing method.  Section 3 describes our experimental analysis method detailed. Some simulation results are presented in Section 4 to show the feasibility of proposed method. Finally, Section 5 concludes the paper.

## 2. Basics of Ordinal Performance

The ordinal performance is concerned with whether the solution belongs to the good enough set. The evaluation criterion is alignment probability. The definition of good enough set and alignment probability is introduced in OO. So, in this section, we briefly overview OO.

### 2.1. Brief Overview of OO

OO was first introduced by Ho et al. in 1992 [15], which has become an important tool for optimizing discrete event dynamic system (DEDS). There are two basic ideas in OO. The first idea is ordinal comparison; that is, “order” is easier to ascertain than “value.” The second idea is goal softening. Instead of only caring about optimal solution, OO is willing to settle for the “good enough” solution.

In OO, Θ is the search space and satisfies |Θ | = *N*. The “good enough” set *G* is defined as the top-*g* of the search space Θ or top *p*% of the search space Θ. It satisfies |*G* | = *g*. Selected set *S* is selected by rule and satisfies |*S* | = *s*. OO can guarantee that *S* contains top-*g* solutions of the search space with a high probability. It is called alignment probability in OO and denoted by *P*_AP_.

### 2.2. Ordinal Performance

The research of solution quality evaluation method transfers from the value performance to the ordinal performance, after the definition of the good enough set, selected set, and alignment probability introduced. Based on this knowledge, Shen et al. [17] proposed evaluation method, called ordinal optimization ruler (OO ruler), using the related knowledge of hypothesis testing. So we can use OO ruler to qualify the ordinal performance of solution. One of the intuitive understandings of OO ruler is that uniform samples are taken out from the whole search space and evaluated with a crude but computationally easy model when applying OO. After ordering via the crude performance estimates, the lined-up uniform samples can be seen as an approximate ruler. By comparing the heuristic design with such a ruler, we can quantify the heuristic design, just as we measure the length of an object with a ruler. If the OO ruler gets from all the solutions, it is an accurate ruler. But this is obviously an ideal situation for practical problems. It is proved that approximate OO ruler is also effective.

**Theorem 1 (see [17])**

If the *k* solution obtained by optimization algorithm is better than *t* solution of selected set obtained by uniform sampling, we can judge that the *k* solution belongs to the top *p*% of the search space Θ at least. And the type II error probability is not larger than *β*_0_. The relation between *s*, *β*_0_, *t*, and *p*% is determined by

(1) $$\begin{array}{c} \overset{t-1}{\underset{j=0}{\sum }}\left(\begin{array}{c} s\\ j \end{array}\right){\left(p\%\right)}^{j}{\left(1-p\%\right)}^{s-j}\leq \beta_{0}, \end{array}$$

where $\left(\begin{array}{c} s\\ j \end{array}\right)$ represents the number of different choices of *s* designed out of *j* distinguished ones.

In the case of given parameters of *s* and *β*_0_, we can get relation between *t* and *p*% through the list method.

For an arbitrary solution obtained by heuristic algorithm, we only need to compare it whether satisfies the conditions of Theorem 1, then we can make the corresponding judgment, so as to realize the evaluation ordinal performance of solution. But OO ruler has a premise. To get OO ruler, uniform sampling for search space is needed. It is also prerequisite for OO. The so-called uniform sampling refers to the same probability of getting arbitrary solution. It is also the reason why the uniform sampling can provide quantitative reference. But, for some problems, it is difficult to achieve uniform sampling, and thus it will not be able to get OO ruler. In addition, the price of getting OO ruler for huge solution space is very high. These two problems limit the application of OO ruler in solution evaluation. However, the introduction of ordinal performance has great inspiration for the research of solution quality evaluation for SI.

## 3. The Framework of Assessment Method

In this section, we take traveling salesman problem (TSP) as an example to describe experimental analysis method of solution quality evaluation.

### 3.1. Sample Characteristics of SI

For SI, the feature of the algorithm itself determines that the sampling method in the search space is not uniform. Especially by the partial reinforcement effect, it makes the algorithm more and more concentrated in certain regions. So it is not suitable for evaluating method directly using OO ruler. In addition, the algorithm produces a large number of feasible solutions. The feasible solution contains the search characteristics of some algorithms and the distribution of the solution space. To obtain the hidden information and its rational utilization through some analysis methods, we need to do some research. It plays an important role in the research of qualtiy evaluation and improving the algorithm performance.

### 3.2. The Framework of Assessment Method

Based on the above analysis, this paper presents a general framework of the quality evaluation method for SI. The framework contains three procedures. First, to get some internal approximate uniform subclass, using cluster method, the solution samples (corresponding to selected subset of OO) were homogeneous processing. Second, discrete probability distribution solution samples of each subclass and the scale relationship of the subclass are estimated in the fitness space. Based on the characteristics of the subclass, the presupposition ratio of the good enough set is distributed to each subclass. Last, alignment probability is calculated according to the model of solution quality evaluation, so as to complete the evaluation of the solution quality.

#### 3.2.1. Uniform Clustering for Nonuniform Samples

According to the characteristics of discrete space, uniform clustering of samples is that obtaining probability of solution is approximating same. Compared with the continuous space, clustering is very different from discrete space. General discrete spatial distance features are defined with the question, and not as the continuous space as a distance to define general way. This makes clustering method based on grid no longer applicable, which is used in continuous space such as density clustering and clustering method based on grid. And the huge solution sample set also limits the use of some special clustering method. Therefore, we need to design a suitable and efficient clustering algorithm based on demand.

Approximate sampling probability is the purpose of clustering. The approximate sampling probability here refers to the neighbor characteristics (including the distance and number of nearest neighbors) consistent approximation. A feasible method for TSP is to calculate the distance between all solution samples. Then clustering is done according to the nearest neighbor statistical feature of each sample distance. But it is only applicable to the small size of the solution sample. Another possible method is that the clustering centers are selected from the best solutions. The distance is calculated between each feasible solution and the cluster center. Then the solution samples are clustered according to the distance. The calculation complexity of this algorithm is low. It is more suitable for clustering large scale solution samples. In the next section, we use this clustering method.

#### 3.2.2. The “Good Enough” Set Decomposition

The solution alignment probability is calculated using a priori ratio of the good enough set (the ration between the good enough set and search space) in OO. The ratio of each kind of the good enough sets is needed to know after clustering. The prior ratio requires decomposing prior ratio of each class. This decomposition has a certain relationship with each class distribution of samples and the class size. Therefore, the distribution characteristics of solution in the fitness value, as well as proportional relation of class size, are needed to estimate.

Estimation of distribution of solution in the fitness value is problem of one-dimensional distribution sequence estimation. The purpose of distribution estimation is to obtain the good enough set distribution. If the fitness value is arranged according to the order from small to large, ordered performance curve (OPC) can be obtained. For the minimization problem, the good enough set is in the first half of the OPC. To obtain a true estimation of the good enough set, you need to consider the types of OPC.

#### 3.2.3. Ordinal Performance Estimation

The original search space after clustering is divided into *l* approximate uniform partition. Search space Θ_*k*_, the good enough set *G*_*i*_, and selected set *S*_*i*_ of each partition and search space Θ, good enough set *G*, and selected set *S* of the original search space have the following correspondence in the collection and base:

(2) Θ1∪Θ2∪⋯∪Θl=Θ,G1∪G2∪⋯∪Gl=G,S1∪S2∪⋯∪Sl=S,N1+N2+⋯+Nl=N,N≡|Θ|, Ni≡|Θi|, ∀i=1,…,l,g1+g2+⋯+gl=g,g≡|G|, gi≡|Gi|, ∀i=1,…,l,s1+s2+⋯+sl=s,s≡|S|, si≡|Si|, ∀i=1,…,l,

where |·| is the base of set ·.

Since the probability of any feasible solution pumped into each subclass is the same, for a sampling result *θ*_*i*_ has

(3) $$\begin{array}{c} P\left(\theta_{s}=\theta_{i}\right)=\frac{1}{N_{i}},\;\forall \theta_{i}\in \Theta_{i}. \end{array}$$

In this paper, we only concern the selected set whether has at least one solution in good enough set. So we can draw the following conclusions:

(4) $$\begin{array}{c} P_{\text{AP}}\left\{\left|S\cap G\right|\geq 1\right\}=1-\overset{l}{\underset{i=1}{\prod }}{\left(1-p_{i}\%\right)}^{s_{i}}. \end{array}$$

#### 3.2.4. Procedures of Assessment Method

The main steps to get the evaluation method by the above analysis are described in Algorithm 1.

## 4. Experimental Results and Analysis

In this section, we take the Hopfield 10-city problem, which is also used in [17], as the example to demonstrate our experimental analysis method. The coordinates of the 10 cities are {(0.4000, 0.4439); (0.2439, 0.1463); (0.1707, 0.2293); (0.2293, 0.7610); (0.5171, 0.9414); (0.8732, 0.6536); (0.6878, 0.5219); (0.8488, 0.3609); (0.6683, 0.2536); (0.6195, 0.2634)}. There is (10−1)! = 362880 solutions in the search space. The best path is [1,4, 5,6, 7,8, 9,10,2, 3] or [1,3, 2,10,9, 8,7, 6,5, 4]. Here we define |*G* | = 0.005*N*. We use two groups of experimental simulation to demonstrate effectiveness of proposed method, where *P*_AP_ is alignment probability. Statistics value represents the alignment probability by our methods. Computational value is the alignment probability, and the error represents the difference of two alignment probabilities.

### 4.1. Evaluation Index

Alignment probability is a measure of whether optimal solution belongs to the good enough set. It is a probability value. Therefore, studying this value has little significance in one experiment. It is needed to do many experiments to study the statistical laws. So, each kind of experiment independently does *K* times. If the optimal of *i* time belongs to the good enough set, let *s*_*i*_ = 1; otherwise *s*_*i*_ = 0. Let *P*_*g*_ be statistical frequency. Then, for *K* times experiment, we have

(5) $$\begin{array}{c} P_{g}=\frac{\sum_{i=1}^{r}s_{i}}{r},\;r=1,2,\ldots ,K. \end{array}$$

From (5), the following can be seen, when *K* tends to infinity:

(6) $$\begin{array}{c} \underset{K\to +\infty }{\lim}{\;P}_{g}=P_{g}^{\prime }, \end{array}$$

where *P*_*g*_′ is the alignment probability value, but it is generally difficult to obtain. In general, we only need to compute the *P*_*g*_ value which may be tested experimentally.

Let ${\overset{-}{P}}_{A}(r)$ be the alignment probability in an experiment by the evaluation method; *P*_*A*_ is average value of ${\overset{-}{P}}_{A}(r)$. Consider

(7) $$\begin{array}{c} P_{A}=\frac{\sum_{i=1}^{r}{\overset{-}{P}}_{A}\left(r\right)}{r},\;r=1,2,\ldots ,K. \end{array}$$

Let *e*_*r*_ be the absolute value of error of *P*_*g*_ and *P*_*A*_; that is,

(8) $$\begin{array}{c} e_{r}=\left|P_{A}-P_{g}\right|. \end{array}$$

In the following experiments, we are using *e*_*r*_ as the standard evaluation index.

### 4.2. Ordinal Performance Evaluation of Nonuniform Sampling

The solution space is sorted according to the fitness values and gets the whole solution space of the sample set, denoted by *Ω*. We deliberately partition the search space into the same two parts *Ω*_1_ and *Ω*_2_. Then we sample, respectively, in parts *Ω*_1_ and *Ω*_2_, respectively. Times are denoted by *n*_1_ and *n*_2_. Then the total number of samplings is *n*. Then

(9) n1n2=ratio,n1+n2=n.

Let *K* = 5000 and *n* = 3000. Because the value of ration can be divided into two cases. One is no less than 1, and the other is less than 1. So, the following points are discussed.

#### 4.2.1. Ratio ≥ 1

**Figure 1:**
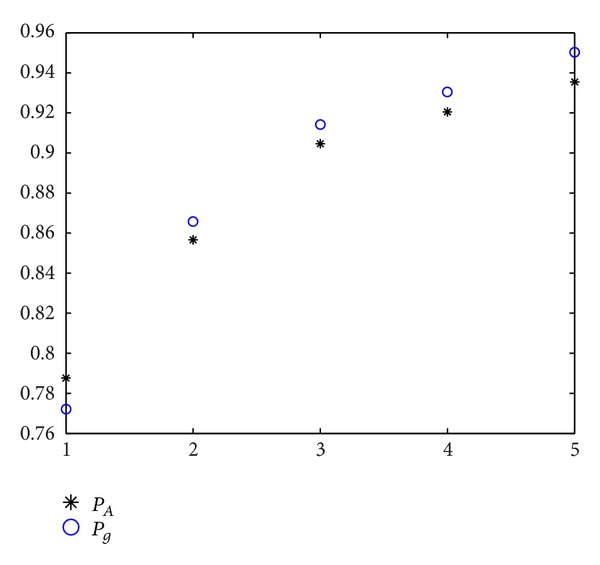
Comparison of two probabilities with ratio ≥ 1.

**Figure 2:**
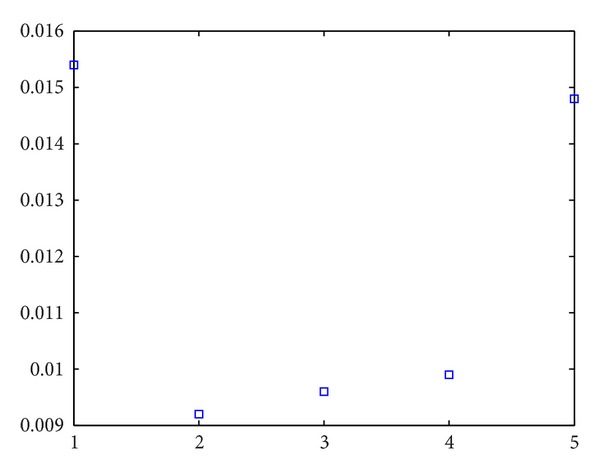
Error of two probabilities with ratio ≥ 1.

This case illustrates the sampling times in area *Ω*_1_ more than in area *Ω*_2_, and the good enough set is in area *Ω*_1_. The experiment results can be seen in Figures 1 and 2. The abscissa is value of ratio. The values from left to right, respectively, are 1, 2, 5, 10, and 100. In Figure 1, we can see that, with the increasing value of ratio, the sampling point in area *Ω*_1_ is increasing. The probability of obtaining the good enough solution increases as the good enough set is in area *Ω*_1_. In addition, except for the case of ratio = 1, *P*_*A*_ is slightly higher than *P*_*g*_. The rest of *P*_*A*_ are lower than *P*_*g*_. The error of two probabilities seen from Figure 2 is lower and no more than 2% generally.

#### 4.2.2. Ratio < 1

**Figure 3:**
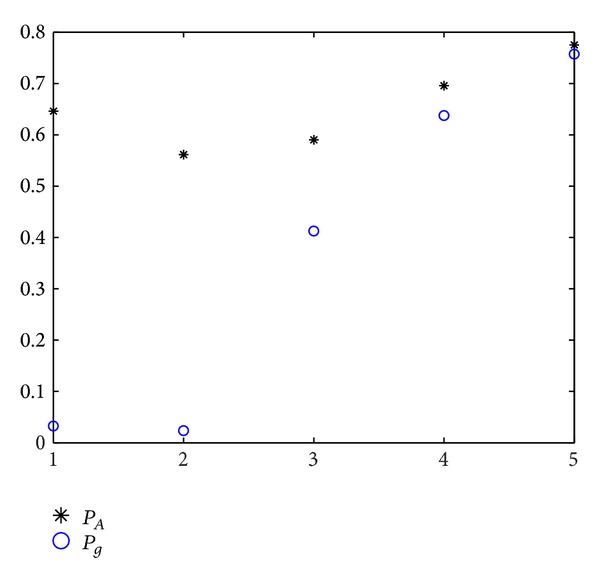
Comparison of two probabilities with ratio < 1.

**Figure 4:**
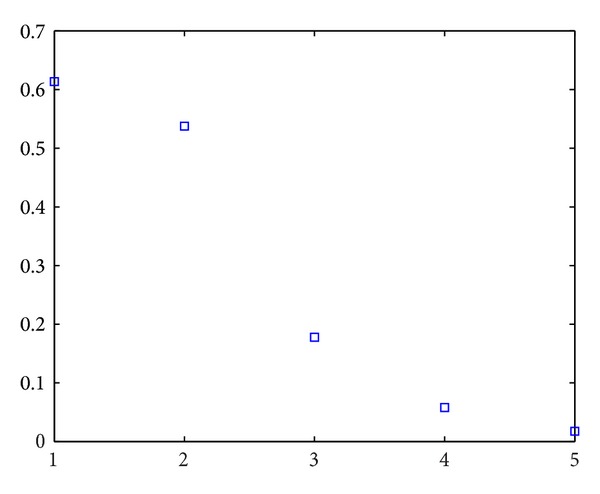
Error of two probabilities with ratio < 1.

This case illustrates the sampling times in area *Ω*_2_ more than in area *Ω*_1_. The experiment results can be seen in Figures 3 and 4. The abscissa is value of ratio. The values from left to right, respectively, are 0.01, 0.1, 0.2, 0.5, and 0.9. In Algorithm 1 and Figure 1, we can see that the error of two probabilities is high. But the error decreases with the ratio increasing.

### 4.3. Ordinal Performance Evaluation of ACO

**Figure 5:**
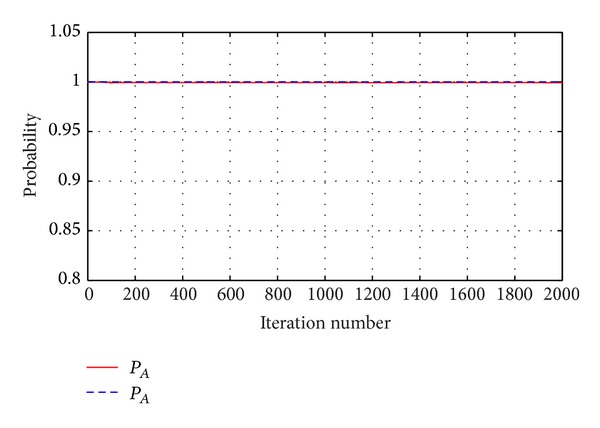
Comparison of two probabilities with ACO.

**Figure 6:**
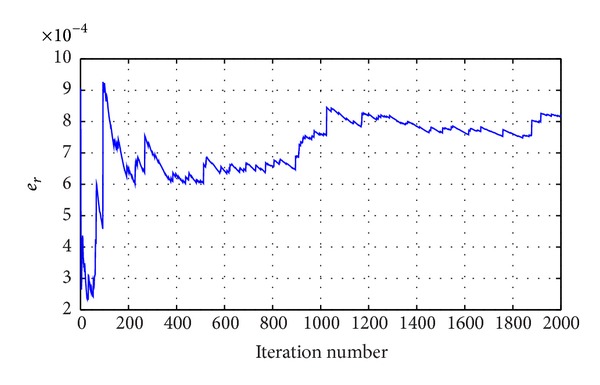
Error of two probabilities with ACO.

Let *K* = 2000; the computational results can be seen from Figures 5 and 6. From Figure 5 we can see that the alignment probability of *P*_*A*_ and *P*_*g*_ is close to 1 and the difference is low. The *P*_*A*_ is slightly lower than *P*_*g*_. It is showed that the evaluation method is conservative. The error range is less than 0.1%. This shows that the calculation result is credible.

In order to further study the relation between the parameters of ant colony algorithm and evaluation results, we focus on the relationship between the maximum number of iterations changes and ant number changes and evaluation of results. The results can be seen from Tables 1 and 2.

First, we study the ant number. The ant number *m* belongs to the set {2,4, 5,8, 10}. From Table 1 we can see that the value of *P*_*A*_ is increasing with the *m* increasing. The error of probability is reducing with the *m* increasing. This shows that the size of solution has some influence on the evaluation method. Second, we study the iteration number *N*_max⁡_ which is selected from the set {10,20,30,50,100,200}. From Table 2 we can see that *P*_*A*_ is much less than *P*_*g*_ when *N*_max⁡_ is 10. But, with *N*_max⁡_ increasing, the error is reducing. The reason is that the information of space is accumulated with *N*_max⁡_ increasing. It is showed that the more the utilization of information of the solution space, the more accurate the result.

### 4.4. Ordinal Performance Evaluation of PSO and AFS

We also do the same comparison for PSO and AFS. The results can be seen from Tables 3 and 4. *m* is particle number in Table 3 and *m* is fish number in Table 4. From Tables 3 and 4 we can see that the value of *P*_*A*_ is increasing with the *m* increasing and the maximum number of iterations *N*_max⁡_. The average solution is also improved. It is showed that the solution quality is effect on *P*_*A*_.

## 5. Conclusion

A solution assessment method of SI is presented in this paper. Based on the analysis of the existing knowledge foundation, combined with the ordinal optimization theory, the ordinal performance is research target to evaluate solution. Then based on the analysis of characteristics of SI algorithms, the framework of evaluation method is given. The detailed steps of the method are presented. Finally, taking the Hopfield 10-city problem as an example, some simulation experiments are done. The experimental results show that the proposed method is feasible.

## Figures and Tables

**Algorithm 1:**
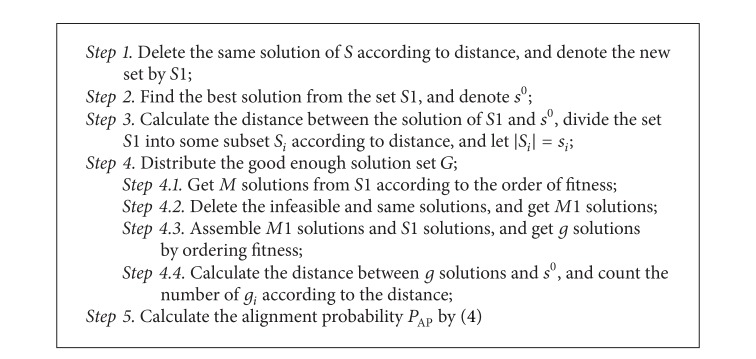
The main steps of assessment method.

**Table 1:** Experimental comparison for ant number *m*.

*m*	Best	Worst	Average	STD	*P*_*A*_	*P*_*g*_	*e*_*r*_
2	2.6907	3.2025	2.7671	0.0840	0.8496	0.9985	0.1489
4	2.6907	2.9689	2.7200	0.0521	0.9626	1.0000	0.0374
5	2.6907	2.9689	2.7111	0.0410	0.9792	1.0000	0.0208
8	2.6907	2.8982	2.6966	0.0222	0.9972	1.0000	0.0028
10	2.6907	2.8982	2.6937	0.0163	0.9992	1.0000	0.0008

The best solution, the worst solution, the average solution quality, and the standard deviation in *K *times running are given.

**Table 2:** Experimental comparison for maximum iteration number.

*N*_ max_	Best	Worst	Average	STD	*P*_*A*_	*P*_*g*_	*e*_*r*_
10	2.6907	3.1879	2.7695	0.0791	0.7750	0.9985	0.2235
20	2.6907	2.9844	2.7138	0.0435	0.9586	1.0000	0.0414
30	2.6907	3.0504	2.7118	0.0440	0.9748	1.0000	0.0252
50	2.6907	2.9390	2.7094	0.0393	0.9803	1.0000	0.0197
80	2.6907	2.9669	2.7073	0.0384	0.9835	1.0000	0.0165
100	2.6907	2.9669	2.7061	0.0358	0.9853	1.0000	0.0147
200	2.6907	2.8982	2.7022	0.0327	0.9909	1.0000	0.0091

**Table 3:** Experimental comparison for PSO.

*m *	*N*_max⁡_	Best	Worst	Average	STD	*P*_*A*_	*P*_*g*_	*e*_*r*_
10	30	2.6907	3.5976	3.0174	0.1543	0.6720	0.7425	0.0713
10	50	2.6907	3.3618	2.9483	0.1310	0.7861	0.8890	0.1029
10	60	2.6907	3.3582	2.9227	0.1222	0.8257	0.9340	0.1083
10	80	2.6907	3.3038	2.8923	0.1104	0.8894	0.9745	0.0851
10	100	2.6907	3.2328	2.8685	0.1029	0.9262	0.9840	0.0578
20	60	2.6907	3.2275	2.8516	0.0947	0.9480	0.9970	0.0490
20	100	2.6907	3.0861	2.8068	0.0736	0.9898	1.0000	0.0102

**Table 4:** Experimental comparison for AFS.

*m *	*N*_max⁡_	Best	Worst	Average	STD	*P*_*A*_	*P*_*g*_	*e*_*r*_
5	50	2.6907	3.1556	2.7439	0.1017	0.7036	0.9985	0.2949
8	50	2.6907	3.0909	2.7108	0.0569	0.8215	1.0000	0.1785
10	50	2.6907	3.0783	2.7034	0.0440	0.8639	1.0000	0.1361
15	50	2.6907	3.0302	2.6929	0.0153	0.9397	1.0000	0.0603
10	80	2.6907	3.0344	2.6970	0.0293	0.8855	1.0000	0.1145
10	100	2.6907	3.0830	2.6956	0.0280	0.8898	1.0000	0.1102
20	80	2.6907	2.7782	2.6909	0.0039	0.9743	1.0000	0.0257
20	100	2.6907	2.7782	2.6909	0.0048	0.9750	1.0000	0.0250
